# Cross-resistance is modular in bacteria–phage interactions

**DOI:** 10.1371/journal.pbio.2006057

**Published:** 2018-10-03

**Authors:** Rosanna C. T. Wright, Ville-Petri Friman, Margaret C. M. Smith, Michael A. Brockhurst

**Affiliations:** 1 Department of Biology, University of York, York, United Kingdom; 2 Department of Animal and Plant Sciences, University of Sheffield, Sheffield, United Kingdom; Wageningen Universiteit en Researchcentrum, Netherlands

## Abstract

Phages shape the structure of natural bacterial communities and can be effective therapeutic agents. Bacterial resistance to phage infection, however, limits the usefulness of phage therapies and could destabilise community structures, especially if individual resistance mutations provide cross-resistance against multiple phages. We currently understand very little about the evolution of cross-resistance in bacteria–phage interactions. Here we show that the network structure of cross-resistance among spontaneous resistance mutants of *Pseudomonas aeruginosa* evolved against each of 27 phages is highly modular. The cross-resistance network contained both symmetric (reciprocal) and asymmetric (nonreciprocal) cross-resistance, forming two cross-resistance modules defined by high within- but low between-module cross-resistance. Mutations conferring cross-resistance within modules targeted either lipopolysaccharide or type IV pilus biosynthesis, suggesting that the modularity of cross-resistance was structured by distinct phage receptors. In contrast, between-module cross-resistance was provided by mutations affecting the alternative sigma factor, RpoN, which controls many lifestyle-associated functions, including motility, biofilm formation, and quorum sensing. Broader cross-resistance range was not associated with higher fitness costs or weaker resistance against the focal phage used to select resistance. However, mutations in *rpoN*, providing between-module cross-resistance, were associated with higher fitness costs than mutations associated with within-module cross-resistance, i.e., in genes encoding either lipopolysaccharide or type IV pilus biosynthesis. The observed structure of cross-resistance predicted both the frequency of resistance mutations and the ability of phage combinations to suppress bacterial growth. These findings suggest that the evolution of cross-resistance is common, is likely to play an important role in the dynamic structure of bacteria–phage communities, and could inform the design principles for phage therapy treatments.

## Author summary

Phage therapy is a promising alternative to antibiotics for treating bacterial infections. Yet as with antibiotics, bacteria readily evolve resistance to phage attack, including cross-resistance that protects against multiple phages at once and so limits the usefulness of phage cocktails. Here we show, using laboratory experimental evolution of resistance against 27 phages in *P*. *aeruginosa*, that cross-resistance is common and determines the ability of phage combinations to suppress bacterial growth. Using whole-genome sequencing, we show that cross-resistance is most common against multiple phages that use the same receptor but that global regulator mutations provide generalist resistance, probably by simultaneously affecting the expression of multiple different phage receptors. Future trials should test if these features of cross-resistance evolution translate to more complex in vivo environments and can therefore be exploited to design more effective phage therapies for the clinic.

## Introduction

Natural microbial communities are comprised of complex networks of species interactions, wherein each species may be engaged in ecological interactions with many other species [1–3]. In this community context, the evolutionary response of a focal species to a given pairwise species interaction can promote an ‘evolutionary cascade’ through the adjacent interacting species [4,5]. For bacteria–phage interaction networks, we expect that the impact of a given phage resistance mutation will depend on the connectivity of that bacterial host within the community network [6] and the degree of cross-resistance provided by the mutation against other phage species in the network [7]. Whereas the statistical structure of interactions in bacteria–phage networks has been well studied [8], the structure and underlying genetic basis of cross-resistance networks remain poorly understood. This considerably limits our ability to predict how cross-resistance evolution affects bacteria–phage communities across different environmental, agricultural, and clinical contexts.

The extent of cross-resistance provided by a given resistance mutation is likely to depend on the genetic correlations between bacterial resistance traits selected by the different phage species. Cross-resistance is likely for cases of positive genetic correlation; for example, binding to shared receptors can cause synergistic pleiotropy between specific resistances—for instance, a mutation in the lipopolysaccharide (LPS) biosynthesis pathway is likely to promote cross-resistance to other phages that also adsorb to LPS [9,10]. In contrast, cross-resistance is less likely if there is antagonistic pleiotropy, in which resistance to one phage increases susceptibility to an alternative phage (for example, through replacement of a clustered regularly interspaced short palindromic repeat [CRISPR] spacer [11]), or no genetic correlation—for instance, if the phages bind to different receptors and accumulation of multiple resistance mutations is therefore required [12]. Because individual resistance mechanisms frequently incur fitness costs by impairing the normal functioning of the molecule acting as the phage receptor, accumulation of multiple resistance mechanisms may be limited by their combined fitness costs, particularly if there is negative epistasis among the fitness costs of resistance mutations [7,13]. Even though pleiotropic costs should limit the evolution of generalist resistance, cross-resistance is commonly observed [14,15]. Most of this evidence is, however, based on relatively simple phage communities, and it is less clear how the range of cross-resistance provided by different resistance mutations is related to the magnitude of fitness costs in more complex bacteria–phage networks.

Understanding the structure of bacterial cross-resistance to phage infection also has important applied implications with relation to phage therapy, i.e., the use of phages as antimicrobials to treat bacterial infections [16]. Phage cocktails (i.e., combinations of different phages) have been shown to delay the evolution of resistance in bacteria, both in vitro [17] and in vivo [18], compared to challenge with a single phage. Effective phage cocktails often contain phages that target different bacterial receptors (for example, [19,20]), and as a result, multiple resistance mutations in different receptor genes are required to provide resistance to all the phages present in the cocktail [21]. The requirement for bacteria to accumulate multiple resistance mutations is thought to enhance the evolutionary robustness of phage cocktails because there is a lower probability of resistance emerging. Furthermore, resistance to multiple phages is likely to be associated with greater fitness costs assuming additivity of fitness costs associated with each resistance mutation [7]. These assumptions may not apply, however, if very generalist cross-resistance is available via a single mutation affecting the expression of multiple phage receptors. This suggests therefore that minimising the potential for cross-resistance could be a key feature of effective phage cocktail design. However, this has not been tested experimentally.

Here we determined the network structure and genetic basis of cross-resistance against a collection of 27 phages infecting the opportunistic human pathogen, *P*. *aeruginosa*. The cross-resistance network contained both symmetric (reciprocal) and asymmetric (nonreciprocal) interactions, forming two cross-resistance modules defined by high within- but low between-module cross-resistance. Within cross-resistance modules, resistance mutations targeted distinct phage receptors, whereas between-module cross-resistance was caused by mutations targeting a global regulator likely to control the expression of multiple phage receptors. The range of cross-resistance provided by a mutation was not correlated to its fitness cost, except that global regulator mutations causing between-module cross-resistance were costlier than mutations causing within-module cross-resistance. Furthermore, the degree and symmetry of cross-resistance predicted the ability of phage combinations to suppress bacterial growth and the frequency of resistance mutations. Together, our data suggest that an understanding of cross-resistance interactions could help to predict the impact of resistance evolution on host–parasite community structure and aid the rational design of therapeutic phage cocktails.

## Results

### Variation in cross-resistance provided by individual resistance mutations

**Fig 1:**
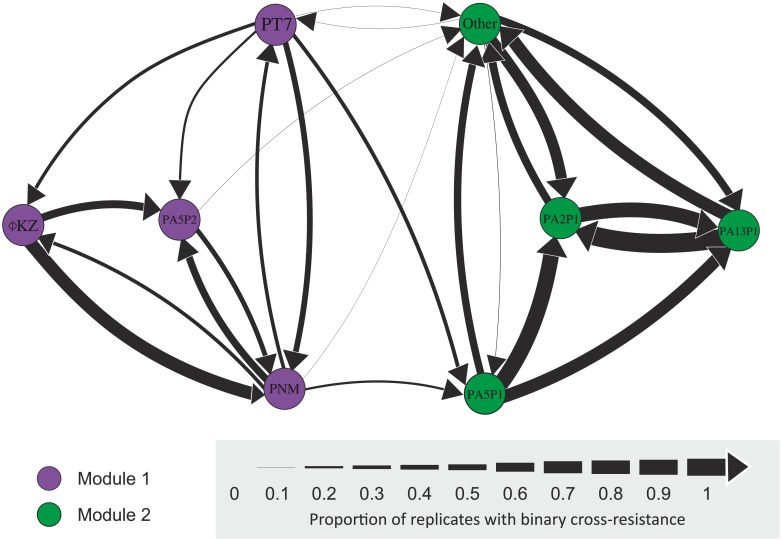
Quantitative cross-resistance network. Each node represents a phage strain, and arrows between nodes show directional CRF between two phages. The line widths are scaled by the proportion of replicate mutants selected against the focal phage (origin node) that have resistance above the binary threshold (S1 Fig) to the second phage (target node). Node colours define resistance modules identified using an ‘edge-betweenness’ algorithm. A subset of 20 phage strains that all showed strong symmetric cross-resistance were grouped together as the node labelled ‘Other’. CRF, cross-resistance frequency.

To determine the extent of cross-resistance, we tested 263 spontaneous resistant mutants of *P*. *aeruginosa* PAO1 selected against each of 27 phages (i.e., 10 resistant mutants were selected against each focal phage; 7 mutants were discarded because of persistent phage contamination) for their ability to resist infection by all other phages (cross-resistance). Resistance was determined by measuring relative bacterial growth (RBG) of each spontaneous mutant in the presence versus absence of each phage where mutants were classified as resistant if their RBG exceeded a binary resistance threshold (RBG = 0.798; calculated as the 95% confidence interval of a normal distribution modelled over the peak of resistance within the complete RBG distribution [S1 Fig; S1 Data]). We observed variation in the pattern and range of cross-resistance among spontaneous resistance mutations (Fig 1; S2 Fig). First, the degree of cross-resistance selected by the different focal phages varied extensively, ranging from conferring resistance against fewer than 10% to up to 80% of all phages (S2 Fig; Kruskal-Wallis χ^2^_26_ = 66.6, *p* < 0.0001). Second, both focal resistance and cross-resistance phenotypes varied considerably between independently evolved resistant mutants selected against the same focal phage (S2 Fig). Together, these results suggest that the magnitude of cross-resistance depends on the focal resistance selected and that multiple resistance mechanisms may exist against the same focal phage, resulting in different levels of cross-resistance between independent replicate mutants.

### Cross-resistance range was not limited by fitness costs or strength of focal resistance

**Fig 2:**
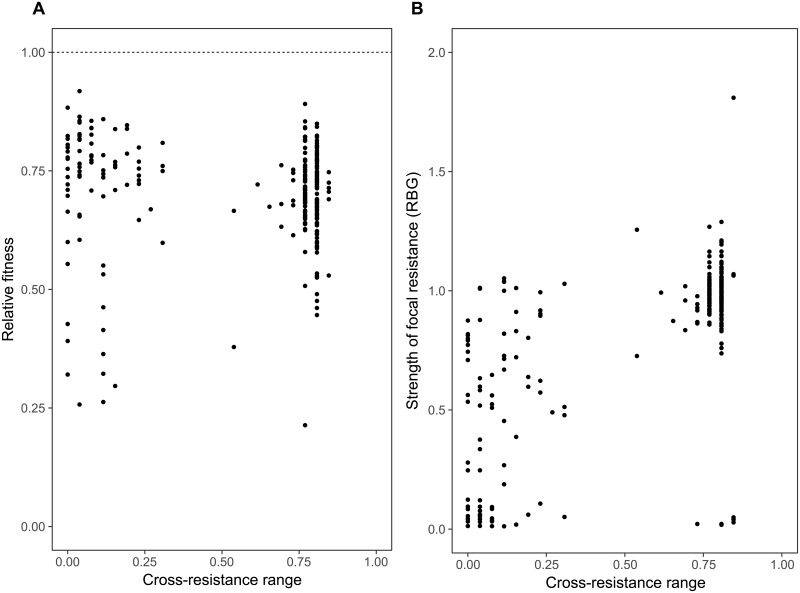
Relationships of cross-resistance range with relative fitness and the strength of focal resistance. Relationship between cross-resistance range (the proportion of nonfocal phages to which the bacterial mutant has resistance above the binary threshold) and relative fitness of spontaneous resistance mutants (A) measured as growth relative to the ancestral strain in phage-free standard media conditions (raw data provided in S2 Data) and (B) the strength of focal resistance, given as growth in the presence of the phage selected against, relative to growth in the absence of phage (RBG). RBG, relative bacterial growth.

The evolution of broad, generalist cross-resistance could be constrained if it was associated with relatively higher costs compared to more specialised resistance or if mutations providing cross-resistance concomitantly provided only weak resistance against the focal phage. In contrast, while all resistance mutations selected against focal phage were costly (Fig 2A; one-sided *t* tests: all *p* < 0.005), we observed no overall relationship between the range of cross-resistance provided by resistance mutations and their associated costs (Fig 2A; linear mixed effects model: t_236_ = −0.655, *p* = 0.513). Moreover, we observed a positive relationship between the strength of focal resistance and the range of cross-resistance provided by resistance mutations (Fig 2B; linear mixed effects model: t_245_ = 15.09, *p* < 0.0001). These results suggest that the evolution of cross-resistance is unlikely to be constrained by trade-offs due to associated fitness costs or the strength of the focal resistance.

### The structure of the cross-resistance network

Network analysis of the directional cross-resistance frequency (CRF) of all pairwise phage combinations produced a cross-resistance network with two distinct modules (Fig 1). Within each module, all possible pairwise phage combinations were connected by some degree of cross-resistance, whereas cross-resistance between the two modules was more limited (narrower arrows between nodes denote low frequency of cross-resistance interactions) and observed only between a small proportion of all the potential phage pairs (8/92, i.e., around 8.7%, Fig 1). Within modules, asymmetric (i.e., nonreciprocal) cross-resistance was more common within module 1, whereas module 2 was dominated by symmetric (i.e., reciprocal) cross-resistance (note that the ‘other’ node in module 2 of Fig 1 contains a subset of 194 mutants providing consistently strong symmetric cross-resistance against 20 phages). The high degree of symmetric cross-resistance observed in module 2 could not be explained simply by the genetic similarity of the focal phages as estimated from their random amplification of polymorphic DNA (RAPD) PCR banding patterns (S4 Fig; S3 Data). Between-module cross-resistance was always asymmetric and typically from module 1 to module 2 (Fig 1). This network structure was robust to the binary threshold value used to classify resistance, although using lower thresholds led to increased numbers of asymmetric connections between modules (S3 Fig). Symmetric cross-resistance is likely to occur when both phages select for similar modifications to a shared receptor, whereas asymmetric cross-resistance could result if phages selected for different modifications to a shared receptor that varied in the extent of disruption or for entirely different resistance mechanisms that varied in the extent of generalism. To study this at the genetic level, we next obtained whole-genome sequences for resistant mutants selected against a subset of 10 focal phages that represented all nodes of the cross-resistance network (S5 Fig).

### Molecular basis of within- and between-module cross-resistance

**Fig 3:**
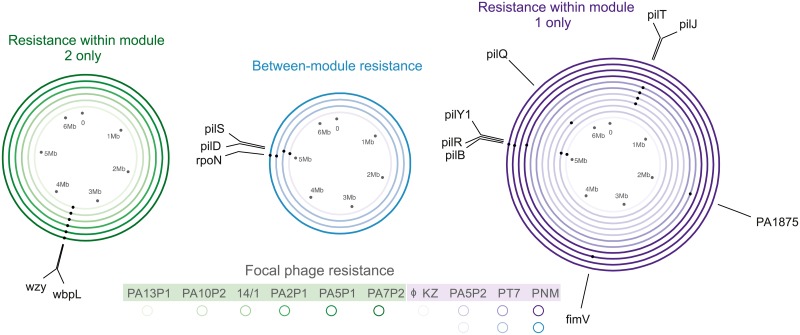
Genetic basis of phage resistance. Circles represent different phage-resistant mutants selected against different focal phages (indicated by the colour shade; see key), and dots on each circle show the position of mutated genes. Colour represents the cross-resistance profile of each sequenced resistant mutant: resistance within module 1 (purple), within module 2 (green), and between modules (generalist resistance, blue).

We obtained whole-genome sequences for 22 independent spontaneous resistant mutants of PAO1 selected against 10 focal phages to identify mutational changes associated with specific phage resistance profiles (Fig 3; S5 Fig). Cross-resistance within module 2 was associated with mutations in LPS biosynthesis genes *wzy* and *wbpL*, whereas cross-resistance within module 1 was associated with mutations in various genes encoding type IV pilus biosynthesis (Fig 3; S1 Table). These included genes encoding mechanical components of the type IV pilus, such as the motor proteins PilB and PilT, and enzymes involved in type IV pilus biosynthesis and assembly such as PilD, a prepilin peptidase. These data confirm that cross-resistance modules were determined by distinct phage adsorption cell-surface receptors—specifically, the LPS for module 2 phages and the type IV pilus for module 1 phages. We confirmed distinct receptor usage by testing the ability of all 27 phages to infect an unpiliated *pilB* transposon mutant: whereas module 1 phages were unable to form plaques on the unpiliated host, module 2 phages infected the unpiliated mutant at the same efficiency as they infected the piliated wild-type PAO1 host (all *p* > 0.1; S6 Fig; S4 Data). Between-module cross-resistance was associated with mutations in genes encoding the transcriptional regulators RpoN and PilS (Fig 3; S1 Table). This suggests that more generalist phage resistance required changes in bacterial gene regulation, which are likely to have broader-scale effects on the bacterial phenotype than mutations affecting structural genes performing steps in biosynthetic pathways. In addition, weaker between-module cross-resistance was associated with a mutation of the prepilin peptidase–encoding gene *pilD* (S7 Fig); here the likely mechanism of between-module cross-resistance is less clear.

### Fitness cost of between-module cross-resistance is gene specific

**Fig 4:**
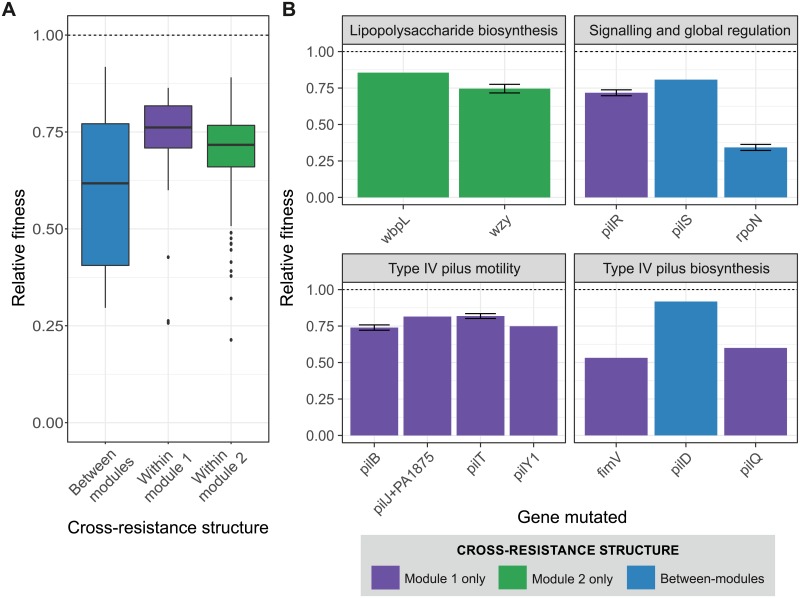
Relative fitness of resistant mutations grouped by cross-resistance type and mutational target. Fitness relative to the phage-susceptible PAO1 ancestor of 263 spontaneous resistant mutants sorted by (A) cross-resistance type or (B) mutated locus. Colours denote cross-resistance type: within module 1 only (purple), within module 2 only (green), and between modules (blue). The dashed line (relative fitness = 1) represents equal fitness to the ancestor.

To test if the different classes of resistance mutations identified by sequencing were associated with different magnitudes of fitness cost, we estimated the fitness of each of the genome-sequenced strains relative to PAO1 in the absence of phage. Between-module cross-resistance was associated with higher fitness costs than within-module cross-resistance (Fig 4A; ANOVA with post hoc Tukey test: module 1, *p* = 0.009; module 2, *p* = 0.015), but this was entirely due to far-higher fitness costs caused by resistance mutations in the *rpoN* gene compared to resistance mutations in either type IV pilus or LPS biosynthesis–associated genes (Fig 4B). Thus, between-module cross-resistance mutations in global regulators that are likely to disrupt many cellular functions are highly costly in the absence of phage, which may limit their long-term survival in bacterial populations.

### Cross-resistance determines the evolution of resistance to phage combinations

We hypothesised that the degree and symmetry of cross-resistance between a pair of phages would predict the frequency of resistance evolution against phage combinations. Specifically, we predicted the highest frequency of resistance mutation would occur against pairs selecting for symmetric cross-resistance, followed by asymmetric cross-resistance, and lowest for no cross-resistance. To test this, we first estimated the frequency of resistance mutations against phage pairs relative to individual phages for all possible combinations of the subset of 10 phages representing all nodes of the cross-resistance network (S5 Fig). One phage (PA5P2) was excluded from further analysis because the absolute resistance mutation frequencies observed against this phage were unfeasibly high (approximately 1.7 × 10^−3^ for PA5P2 alone; S8 Fig). This could possibly indicate that a physiological mechanism of resistance against PA5P2 infection exists, in addition to the LPS-associated mutational mechanism observed in the sequenced resistant clone (Fig 3; S1 Table).

**Fig 5:**
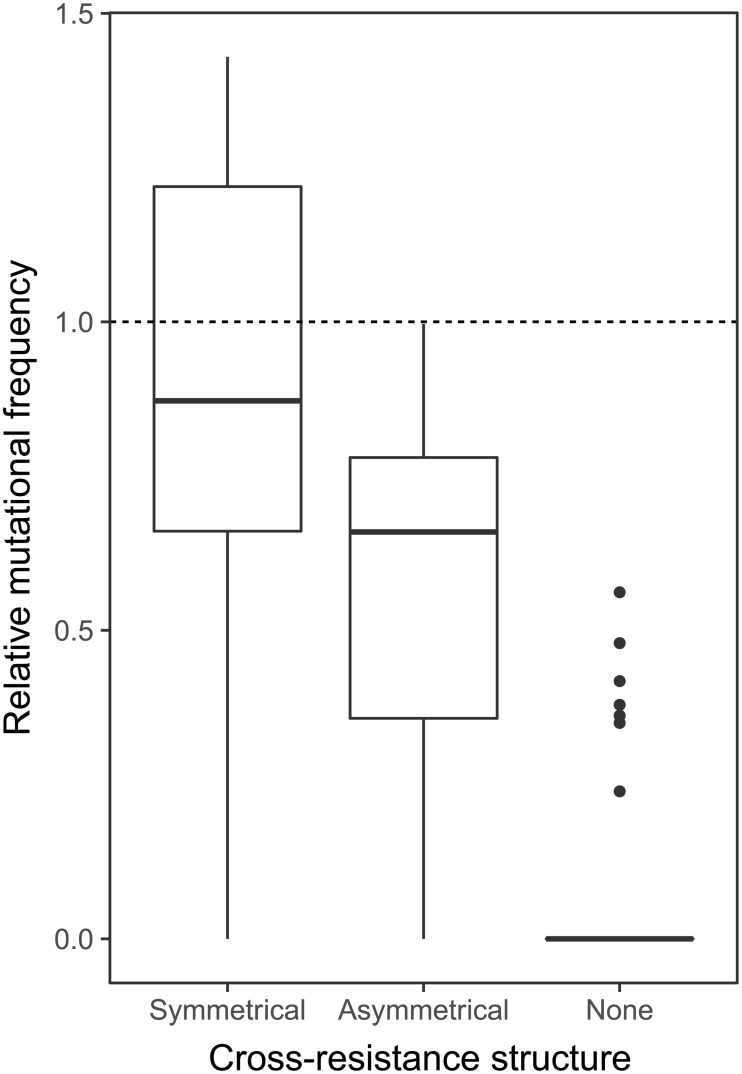
Relative mutational frequency for resistance against phage pairs exerting symmetrical, asymmetrical, or no cross-resistance. Relative mutational frequency compares the frequency of resistance mutations against phage pairs to the geometric mean of mutational frequency against each individual phage. Dashed line represents equal frequency of resistance mutations against a phage pair versus the constituent phages individually. Raw data are provided in S5 Data.

We found a positive relationship between the cross-resistance index (CRI; a nondirectional measure of cross-resistance) and the relative mutation frequency (linear regression R^2^ = 0.280, F_1,106_ = 42.7, *p* < 0.0001). Moreover, consistent with our hypothesis, the relative mutation frequency was highest for phage pairs that selected for symmetric cross-resistance (Fig 5; symmetrical versus asymmetrical *p* = 0.001; symmetrical versus none *p* < 0.0001) and lowest for phage pairs that selected no cross-resistance (Fig 5; asymmetrical versus none *p* < 0.0001). Thus, cross-resistance per se increased the frequency of resistance mutations, with the effect being strongest when cross-resistance was symmetric.

Consistent with the observed resistance mutation frequencies, phage pairs that selected no cross-resistance suppressed the growth of PAO1 most effectively during 24 h, whereas the effect of phage pairs that selected symmetric cross-resistance rarely differed from the best-performing individual phage (S9 Fig; S6 Data). Two phages did not conform to this pattern: Firstly, phage PA10P2 alone was sufficient to completely suppress bacterial growth, and all pairs including this phage were highly effective regardless of the symmetry of the cross-resistance. Secondly, phage pairs containing PA2P1 consistently performed poorly, irrespective of the symmetry of cross-resistance. These results suggest that the structure of cross-resistance predicts the performance of phage combinations but that strong phage identity effects can override this by either increasing or decreasing the efficacy of a phage combination more than expected by cross-resistance alone.

## Discussion

We analysed the network structure and underlying genetic basis of cross-resistance evolution in the bacterium *P*. *aeruginosa* PAO1 selected against 27 phages. Our data show that spontaneous resistance mutations against a focal phage commonly provide cross-resistance against other phages. The cross-resistance network was highly modular, containing two cross-resistance modules with high within- but weak between-module cross-resistance interactions. At the genetic level, cross-resistance modules were defined by shared mutational targets encoding biosynthesis of phage adsorption receptors (LPS or type IV pilus, respectively), whereas between-module cross-resistance was associated with mutations targeting regulatory genes. The strength, direction, and symmetry of cross-resistance between phage pairs predicted both the frequency of resistance mutation and the efficacy with which the phage pair suppressed bacterial growth: the highest-performing phage combinations were those that selected no cross-resistance, whereas the lowest-performing combinations selected symmetric cross-resistance. Taken together, these data suggest that cross-resistance will commonly shape the dynamic structure of bacteria–phage communities and that it is likely to be an important predictor of the robustness of phage therapy to resistance evolution. Further experiments will be required, however, to test whether cross-resistance predicts the efficacy of phage cocktails in more complex in vivo environments.

Our finding that cross-resistance was common for our phage collection suggests that resistance evolution events may frequently disrupt the structure of bacteria–phage interaction networks. Cross-resistance evolution has the effect of reducing connectance at the whole-community level more than would be expected if all interactions were strictly pairwise. The effect of connectance on community stability is dependent on underlying species interaction network architecture; reduced connectance can increase stability in trophic networks by enhancing modularity but may reduce stability in networks with nested structures [22]. Bacteria–phage networks typically have a nested-modular network structure [23,24]; thus, the impact of cross-resistance-mediated reduced connectance is likely to depend on precisely where in the network cross-resistance interactions occur. It seems reasonable to assume that modularity in bacteria–phage interaction networks may be caused by the same mechanism that causes cross-resistance modularity—namely, shared phage receptors. If this assumption is correct, then cross-resistance evolution may frequently lead to the collapse of isolated nested modules without destabilising the broader interaction network. Resistance in our experiments arose largely through mutations affecting the expression or biosynthesis of surface receptors. Notably, PAO1 lacks a CRISPR system, and it is likely that CRISPR-mediated phage resistance would cause cross-resistance to only very closely related phages that are sequence identical for the genomic region targeted by the newly integrated spacer. In systems where both resistance mechanisms occur, the mechanism by which resistance evolves is thought to depend on ecological conditions, with surface receptor modification favoured in high-resource environments with high phage densities [25], suggesting that in such systems, the structure of cross-resistance evolution may be highly ecologically contingent.

While resistance mutations were costly, we found no overall relationship between the range of cross-resistance provided by resistance mutations and their associated fitness costs, suggesting that the evolution of cross-resistance is unlikely to be constrained by fitness trade-offs except for rare cases of very generalist between-module cross-resistance (Fig 4B). This finding is somewhat surprising, since previous studies of pairwise bacteria–phage interactions and interactions between bacteria and multiple phage species have reported that broader resistance ranges are associated with higher costs [7,26]. In contrast to these studies, however, we did not allow broad resistance ranges to evolve via accumulation of multiple sequential mutations but instead measured the effects of single spontaneous resistance mutations on cross-resistance. It seems likely therefore that higher costs of generalist phage resistance described previously arise from negative epistasis between multiple resistance mutations rather than from inherent costs of cross-resistance itself. At a community level, cross-resistance could limit the ability of phages to maintain bacterial diversity via density dependent killing [27]. This could be mitigated by migration between local communities, promoting invasion of novel phages to which resistance is absent in the local community, or through phage counteradaptation. For example, phages have been shown to switch hosts in multihost environments [4] and expand host range through spontaneous mutation [28,29]. An important caveat is that fitness costs measured in simple lab environments are likely to underestimate the full extent of the pleiotropic effects of resistance mutations in more complex in vivo environments relevant to phage therapy. For example, loss of the type IV pilus is likely to be highly detrimental in vivo where type IV pilus–mediated motility, attachment, and biofilm formation play important roles in pathogenesis [30].

The modular structure of the cross-resistance network was determined by the shared phage receptors modified by resistance mutations. Within cross-resistance modules, mutations targeted biosynthesis of specific surface receptor targets for phage binding (S1 Table), specifically the type IV pilus in module 1 or the LPS biosynthesis in module 2. Resistance to the same phage could be provided by mutations to different genes in the same pathway. For example, mutations selected against phage PA5P2 affected PilD (a peptidase that processes prepilins; [31]), PilQ (which is involved in assembly and transport; [32]), and PilT (a motor protein that powers pilus retraction; [33]). By contrast, mutations that provided very generalist between-module resistance targeted the regulatory genes *rpoN* and *pilS*. RpoN is an alternative sigma factor that regulates transcription of approximately 700 genes, influencing a diverse range of functions, including motility (via both type IV pilus–and flagella-associated genes), quorum sensing, mucoidy, and biofilm formation [34]. PilS is part of a two-component regulatory system that promotes pilus expression by activating RpoN [35]. The diverse regulatory function of RpoN makes it difficult to identify the specific mechanism of generalist phage resistance. However, RpoN regulation of LPS-associated genes has been identified in *P*. *aeruginosa* (*rfaD*; [34]) and shown to directly influence LPS expression in *Salmonella enterica* (via *rfaH*; [36,37]). It is possible, therefore, that global regulatory mutations affecting the expression of multiple phage receptors could be typical for very generalist phage resistance. Crucially, only less than 10% of resistance mutants possessed generalist cross-resistance against both LPS and pilus binding phages, which suggest that these mutations are rarer. This is intuitive, since there are many more mutational targets in each biosynthesis pathway compared to the single copy of the *rpoN* gene. Moreover, the evolutionary success of global regulator-mediated resistance may be limited by the extensive pleiotropic effects of such mutations on the bacterial phenotype. Consistent with this, *rpoN* mutants suffered the greatest impairment in growth rate of all the observed resistance mutations (Fig 4B). Between-module cross-resistance could be achieved at lower cost through mutations affecting *pilS*, which were no more costly than other resistance mutations in type IV pilus–associated genes, suggesting that loss of PilS-mediated activation of RpoN may have been less disruptive to the cell than loss of RpoN itself. Because RpoN controls expression of important virulence-related functions such as quorum sensing and biofilm formation, phage combinations that select for these mutations may concomitantly drive reduced virulence.

We observed that asymmetric cross-resistance was common within our cross-resistance network. Reducing the threshold used to define resistance further increased asymmetric connections between modules (S3 Fig), suggesting that asymmetric cross-resistance may often be rather weak. While the mechanistic basis of symmetric cross-resistance appears conceptually straightforward—the two phages select for similar modifications to and/or loss of a shared receptor (for instance, via LPS modification [15])—the situation is likely to be more complex for asymmetric cross-resistance. We propose two potential routes to asymmetric cross-resistance: First, phages may select qualitatively different mechanisms of resistance offering different degrees of generality; for example, one phage may select for mucoidy [38,39], masking a number of different phage receptors thus providing cross-resistance, whereas the other phage may select for modification of a specific receptor only and limited cross-resistance. Second, resistance mutations for each phage may target different points in a biosynthesis pathway, such that mutations affecting the start of the pathway will provide greater cross-resistance than those affecting targets downstream [40]. Consistent with the latter mechanism, within module 1, the observed mutations in *rpoN*, which are likely to result in an unpiliated phenotype, provided complete cross-resistance within module 1, whereas mutations to genes lower down the pilus biosynthesis pathway (for instance, *pilB* and *pilT* encoding motor proteins that control extension and retraction of the pilus respectively; [33]) provided cross-resistance to only half of the module 1 phage. Understanding the mechanistic basis of cross-resistance in general, and the symmetry of cross-resistance in particular, should be a target of future research.

Our findings show that cross-resistance and its symmetry predict the efficacy of phage combinations both in terms of the frequency of resistance mutation and the efficiency of suppression of bacterial growth. Frequencies of resistance mutation were the highest for phage pairs with symmetric cross-resistance and the lowest for phage pairs that showed no cross-resistance, suggesting either multistep mutational changes or rarer generalist resistance mutations were required in the latter scenario. Consequently, phage pairs that exerted no cross-resistance often completely supressed bacterial growth, whereas failure to supress bacterial growth was more common for phage pairs that promoted some degree of cross-resistance evolution. Our analysis also identified individual phage strains that increased (or decreased) the performance of phage combinations more than predicted by cross-resistance alone. Although we observed an overall positive association between the strength of focal resistance and the strength of cross-resistance, in some cases, focal resistance caused by a mutation was quantitatively weaker than the cross-resistance(s) or undetectable. Since spontaneous resistant mutants have not had the opportunity to specialise their resistance against the focal phage, it is perhaps unsurprising that stronger cross-resistance can arise by chance. More puzzling are the cases of undetectable focal resistance despite there being observable cross-resistance and a resistance mutation identified in the genome sequence. This phenomenon was limited to particular phages: ϕKZ (*pilR* mutation), PA5P1 (*wzy* mutation), and PT7 (*pilS*, *pilT*, and *pilJ* mutations). Although we do not understand the mechanism underlying missing focal resistance, it is possible that this could be caused by extremely high rates of phage evolution to overcome the resistance mutation during the RBG assay, or particular phages being able to use an alternative surface receptor [41]. This suggests that other properties of phage life history are likely to affect their usefulness in phage combinations and that predictions based on cross-resistance networks could be sensitive to strong phage identity effects.

*P*. *aeruginosa* is a common cause of opportunistic infections, frequently of burn wounds, and is also the major pathogen associated with chronic infections of the cystic fibrosis (CF) airway [42–45]. High-level antibiotic resistance frequently makes *P*. *aeruginosa* CF infections nonresponsive to antibiotic treatments and consequently very difficult to eradicate [46]. As a result, phage therapy has been suggested as a potential alternative or complementary treatment [47–49]. Phage therapy has shown promising results against *P*. *aeruginosa* in both artificial CF lung sputum–like environments and murine models [50]. However, clinical trials of phage therapy on *P*. *aeruginosa*–colonised burn wounds have proved inconclusive thus far [51,52]. Our results suggest that unknown patterns of cross-resistance selected by the phages used in therapeutic cocktails could account for some degree of variation in the efficacy of phage therapies in these studies. Using combinations of phages that do not select for cross-resistance could potentially improve the efficacy and robustness of phage cocktails, increasing their ability to suppress bacterial growth by limiting resistance evolution. While caution is required when making inferences from simple lab experiments to far more complex in vivo environments, our data suggest that analysis of cross-resistance networks could aid the design process for improved therapeutic phage cocktails and warrants future in vivo experimental tests.

## Materials and methods

### Study organisms

A total of 27 different phage strains (S2 Table) that were able to infect *P*. *aeruginosa* PA01 strain were used. Four of the phage strains have been previously characterised and are known to be phylogenetically, structurally, and serologically different [53,54] and promote bacterial resistance evolution to differing degrees [14]. The remaining 23 phage strains were isolated at the same time and location (sewage water treatment facility, Jyväskylä, Finland; for isolation protocols and infectivity ranges, see [55]). Sequence data are available for only two of these strains.

### Characterisation of phage genetic relatedness

The genetic similarity of all phage strains was characterised using RAPD PCR that uses a set of primers (S3 Table) to amplify random sections of DNA, giving a unique PCR banding pattern for each distinct phage genotype. Phage DNA was extracted using the QIAamp DNA Mini Kit (Qiagen, Hilden, Germany), and then a PCR was performed on each phage DNA sample (27 phages), with each primer (0.8 μm final primer concentration; S3 Table) under the following conditions: 4 initial cycles of 94 °C for 45 s, 30 °C for 120 s, and 72 °C for 60 s, followed by 25 cycles of 94 °C for 5 s, 30 °C for 30 s, and 72 °C for 30 s, ending with 72 °C for 10 min. PCR products were run on 1% Agarose gels for 30 min at 200 V. A difference matrix based on these banding patterns (i.e., the proportion of bands that two phages do not have in common) was used to make a neighbour-joining tree (R package ‘ape’, [56]).

### Culture conditions

All bacterial cultures were grown in 6 ml King’s media B (KB) in 30-ml glass microcosms with loose-fitting plastic lids and incubated at 37 °C with orbital shaking (200 rpm). Phage cultures were prepared by inoculating frozen stocks into 30-ml microcosms containing 6 ml KB with 60 μl of PA01 overnight culture (approximately 10^9^ cells ml^−1^). Following overnight incubation at 37 °C, shaken, phage stocks were isolated by filtration (0.22 μm) and stored at 4 °C.

### Selecting spontaneous phage-resistant mutants

To select spontaneous phage-resistant mutants, a modified fluctuation test was used [57]. To establish 135 independent subpopulations of PAO1, we selected a single colony and incubated for 8 h before diluting by 1 in 10 into individual wells of 96-well microplates containing 200 μl of KB medium. Following overnight incubation, each of the bacterial populations was exposed to one of the 27 phages. Specifically, the overnight bacterial cultures were diluted by 10^−2^ directly into 200 μl of a phage stock solution, giving a multiplicity of infection of approximately 100 phage particles per bacterial cell and 5 independent bacterial populations per phage strain. From each bacteria–phage mixture, 100 μl was plated on KB solid agar and incubated overnight. Two colonies per plate were then restreaked onto KB agar plates and grown overnight to remove phage particles. We then picked a single colony from each streak plate to give 10 resistant mutants per phage strain (270 in total), which were then grown overnight in KB before preparing glycerol stocks (40% glycerol) and storing at −80 °C. These overnight cultures were also filter sterilised (0.22 μm) and plated on KB soft agar (0.8%) containing ancestral PA01 to check for any remaining phage particles. If phages were detected, phage-free stocks were created by restreaking the resistant mutant from glycerol stocks and repeating the last step. For seven replicates, we were unable to isolate phage-free stocks; therefore, these replicates were excluded from the analysis, leaving 263 resistant mutants in total.

### Cross-resistance assays

To assess the extent of cross-resistance conferred by resistance against individual phage strains, all 263 resistance mutants were assayed against each of the 27 phage strains individually. Cross-resistance assays were performed in 96-well microplates (final volume of 150 μl) in KB media, at an approximate multiplicity of infection of 10 phage particles per bacterial cell. The RBG [58] was calculated by comparing absorbance readings (600 nm at *t* = 0 and *t* = 8 h) in the presence and absence of phage (Eq 1). RBG is a quantitative measure of bacterial resistance in which 1 indicates equal growth in the presence and absence of phage (i.e., complete resistance), and 0 indicates zero growth (i.e., complete susceptibility).

For phage *i*, bacteria *j*:

$${RBG}_{ij}=\frac{{\left[{Abs}_{600}(t=8\;h)-{Abs}_{600}(t=0\;h)\right]}_{ij}}{{\left[{Abs}_{600}(t=8\;h)-{Abs}_{600}(t=0\;h)\right]}_{controlj}}$$ (1)

Cross-resistance range describes the proportion of phages to which each resistant bacterial replicate mutant is resistant to, using a resistance threshold (RBG = 0.798) calculated as the 95% confidence interval of a normal distribution modelled over the peak of resistance within the complete RBG distribution (S1 Fig).

### Measuring the fitness costs associated with cross-resistance

To determine the fitness costs associated with different cross-resistance profiles, the growth of all resistant mutants was measured in the absence of phage and compared to growth of the ancestral PA01 strain. Bacterial cultures were inoculated directly from glycerol stocks into 150 μl of KB media in 96-well microplates. Absorbance at 600 nm was measured every 30 min for 24 h (37 °C, shaken) to create a growth curve for each resistant mutant. Because of variation in the type of fitness costs observed (i.e., increased lag, reduced maximum OD, and reduced growth rate; S10 Fig), we used the integral of each growth curve as a combined measure of the effect of resistance mutations on bacterial growth. The integral of the growth curve correlates well with each of the other growth parameters (S11 and S12 Figs). The integral of each growth curve gives the total growth for each bacterial strain; dividing this value by the average integral for the ancestral PA01 strain gives an estimate of relative fitness.

### Determining the cross-resistance network

Cross-resistance interactions between two phage strains can be quantified as the proportion of resistance mutants screened against one phage that display cross-resistance (RBG above 0.798) against the second phage, giving a directional metric of cross-resistance strength (CRF). To enable comparison of pairwise phage interactions, a nondirectional CRI was used as the mean of the two directional CRF values.

CRF can be used to construct an interaction network showing all directional cross-resistance interactions within a phage community. Firstly, an adjacency matrix is produced, containing directional CRF values for all possible phage pairs. The R package ‘igraph’ [59] was used to convert the adjacency matrix into a network graph (a list of all the realised links in the network and their associated weights; graph.adjacency function), which can then be plotted (plot.igraph function) as a directional weighted network. In the network, each node represents a single phage strain, and the directional connections are weighted by CRF, showing the frequency of cross-resistance against each phage. A community-detection algorithm (cluster_edge_betweenness function in the ‘igraph’ R package [59]) was used to identify the phage strains within the cross-resistance network that formed modules. This edge-betweenness algorithm [60,61] finds the optimum community structure of a given network by assigning a ‘betweenness’ value to every link in the network based on the frequency with which the link is used to create pathways between all possible pairs in the network. High ‘betweenness’ values indicate links between poorly connected modules. By removing these links in a stepwise manner (recalculating ‘betweenness’ values each time), the algorithm can define modules within the community. The subnetwork of the 10 phages used in further analysis was extracted from this full network.

### Quantifying the frequency of phage resistance mutations

Modified fluctuation tests [57] were used to estimate bacterial mutation frequencies against either individual phage strains or combinations of two phage strains. Three microcosms were inoculated from single colonies of the ancestral PA01 strain. After overnight incubation, each microcosm was subcultured into 55 wells of a 96-well microplate, diluting by 10^−1^ to a final volume of 200 μl, and then allowed to grow overnight at 37 °C in a static incubator. Concurrently, stock solutions of 10 phage strains (a subset representing each node within the CRF network; S5 Fig) were prepared (as above). Phage combinations were assembled, consisting of each phage alone (100 μl) and 1:1 mixtures of each possible phage pair (final volume 100 μl), to give 55 different phage combinations. One independent 200 μl PA01 culture from each of the three replicate microplates was then diluted 100-fold into each of the phage solutions, giving a multiplicity of infection of approximately 100 phage particles per bacterial cell, and incubated for 30 min at 37 °C in a static incubator.

Initial bacterial cell density was estimated by plating serial dilutions of 6 random 200 μl PA01 cultures per replicate microplate. The number of phage-resistant spontaneous mutants was then calculated by plating 60 μl of each bacteria–phage mixture onto solid KB agar to give colony-forming units per ml (CFU/ml). The ratio of phage-resistant mutants to initial bacterial cell density provides an estimate of the mutational frequency (MF, Eq 2) against each phage combination, and then comparison to the individual phage strains gives relative mutational frequency (RMF, Eq 3).

For phage suspension *i*, bacteria *j*:

$${MF}_{i}=\frac{{\left[CFU/ml\right]}_{ij}}{{\left[CFU/ml\right]}_{controlj}}$$ (2)

For phage pair *i*1 and *i*2,

$$RMF=\frac{{MF}_{i1i2}}{\sqrt{{MF}_{i1}.{MF}_{i2}}}$$ (3)

### Suppression of bacterial growth by phage combinations

To determine the ability of phage combinations to suppress growth of the ancestral PA01 strain, bacterial growth was measured over 24 h in the presence of individual phage strains and all possible pairwise phage combinations of 10 phage strains. This phage subset contains all 4 phages from module 1 and 6 module 2 phages (S5 Fig) and is comprised of asymmetric (*N* = 11) and symmetric (*N* = 13) cross-resistance interactions, as well as pairwise interactions, which promote no cross-resistance (*N* = 21). Individual colonies of ancestral PA01 were inoculated into KB media and, following overnight incubation, were transferred to fresh KB media in 96-well microplates, diluting 10-fold. Phage suspensions were added at an approximate multiplicity of infection of 100 for both individual phage and pairwise phage combination treatments (prepared from phage stock solutions with a 1:1 ratio). Absorbance at 600 nm was measured every 30 min for 24 h during incubation at 37 °C with regular orbital shaking to produce growth curves for PA01 in the presence of each individual phage strain and all possible pairwise phage combinations within the phage subset (S5 Fig), each replicated three times.

### Sequence analysis

To assess the genetic basis of cross-resistance, we randomly chose one resistant mutant screened against each phage within the cross-resistance subnetwork (S5 Fig), along with additional mutants representing symmetrical and asymmetrical cross-resistance profiles within resistance module 1, to be sequenced (22 independent spontaneous mutants in total). Bacteria were sequenced using the Illumina MiSeq platform, followed by bioinformatic analysis as follows: reads were aligned using Burrows-Wheeler Aligner [62], SNPs and small indel variants were called by GATK HaplotypeCaller [63], and then gene information was added using SNPeff [64]. Variants were filtered for quality by the following parameters: coverage of >20 reads per base pair and frequency of alternative allele in >80% of reads. The quality of each variant was further assessed visually using an alignment viewer (igv; [65]). Additionally, called variants occurring in all 22 sequenced mutants were discarded, as these represent mutations present in the ancestral PAO1 compared to the available reference strain used (accession ID AE004091). All sequence data have been uploaded to the European Nucleotide Archive (accession ID PRJEB27828).

### Confirmation of phage surface receptor targets

To confirm that distinct cell surface receptors are required for infection by module 1 phages compared to module 2, we tested the ability of all 27 phage strains to infect a *pilB* transposon mutant (PW8623 pilB-G07::ISlacZ/hah; *P*. *aeruginosa* Two Allele Library) versus wild-type PAO1. Bacteria lawns were prepared as follows: three colonies were selected for each bacterial strain, inoculated into KB media (6 ml), and grown overnight at 37 °C, shaken; 200 μl of each culture was added to 12 ml of soft KB agar (0.6% agar) and poured over set standard KB agar (1.2% agar) in a 120-mm square petri dish to form a bacterial lawn. Filtered phage stocks were serially diluted, and each dilution was spot plated (5 μl) onto a lawn of each bacteria. Plates were incubated at 37 °C for 24 h, and then phage plaques were counted and density calculated as plaque-forming units per ml.

### Statistical analysis

All analysis was conducted in R [66]. Resistant mutants originating from the same subpopulation were treated as paired replicates to prevent pseudoreplication. This means for 263 resistant mutants, we have 133 independent replicates. Variation in cross-resistance range between different focal phages was analysed using the nonparametric Kruskal-Wallis test, after averaging within subpopulations. To test for associations between cross-resistance range and focal resistance or relative fitness, linear mixed effects models (R package ‘lmerTest’ [67]) were used, with subpopulation included as a random effect. Variation in relative fitness between mutants with different network-level cross-resistance (i.e., within/between modules) was analysed using a one-way ANOVA followed by post hoc testing (Tukey test). Comparison of phage densities between transposon mutant (*pilB*) and wild-type hosts was performed using a linear mixed effects model, with bacteria and phage treated as interacting fixed effects. Statistical analysis of the effect of cross-resistance interaction type (i.e., symmetric/asymmetric) on RMF data was performed using the Kruskal-Wallis test, followed by post hoc testing (pairwise Mann-Whitney U) to compare interaction types.

## Supporting information

S1 Fig RBG distribution and definition of the binary resistance threshold. The frequency histogram shows the RBG values for 263 resistant mutants against all 27 phages. The blue curve shows the normal distribution of the resistance peak (RBG = 1). The threshold of binary resistance was calculated as the 95% confidence interval of the normal distribution (blue dashed line; RBG = 0.798). RBG, relative bacterial growth.
(EPS)

S2 Fig Bacteria–phage infection network. Infection network showing resistance profiles of spontaneous mutants selected against individual phage. Spontaneous PA01 mutants are grouped so that each row represents up to 10 replicates selected against the same phage. Each column denotes individual phage strains that mutants were individually challenged against. Colour shading corresponds to the proportion of replicates that were susceptible to the corresponding phage (see key).
(EPS)

S3 Fig Cross-resistance network structure at different binary resistance thresholds. Number and type of links between phage pairs within and between each cross-resistance module for different values of the binary threshold of resistance.
(EPS)

S4 Fig Characterisation of phage genetic diversity. (A) Banding patterns produced by RAPD PCR analysis of 27 phages (columns) by the molecular weight of PCR products from 9 different primers (rows; S3 Table). (B) Difference matrix summarising the dissimilarity of banding patterns between phages (i.e., 1 − proportion of bands in common). (C) Neighbour-joining tree produced from the phage dissimilarity matrix. RAPD, random amplified polymorphic DNA.
(EPS)

S5 Fig Cross-resistance subnetwork. A subset of the cross-resistance network showing all pairwise cross-resistance interactions between the 10 phage strains used in the mutational frequency experiment and the suppression of bacterial growth assays.
(EPS)

S6 Fig Phage infection of PAO1 versus a *pilB* transposon mutant. Plaque-forming units per ml when plated on the piliated wild-type (black; ‘Ancestor’) and the unpiliated *pilB* transposon mutant (grey).
(EPS)

S7 Fig Cross-resistance profiles of sequenced resistant mutants. Bacteria–phage infection network showing sequenced resistant mutants where gene location of SNPs is identified (rows), against 27 phage strains (columns). Strength of resistance (RBG) scales from complete resistance (white) to complete susceptibility (dark blue). RBG, relative bacterial growth.
(EPS)

S8 Fig Absolute frequencies of resistance mutations against phage pairs. Frequency of resistance mutations against single phage (+) and phage pairs coloured by cross-resistance type associated with each phage pair (blue—no cross resistance; green—asymmetrical cross-resistance; red—symmetrical cross-resistance).
(EPS)

S9 Fig Suppression of bacterial growth by phage monocultures and pairwise phage combinations. Bacterial density was measured as OD (600 nm, y-axis) over 24 h (x-axis) in the presence of single phages (white background) or two phages (grey backgrounds). Dark grey background denotes phage pairs that exert symmetric cross-resistance, mid grey for phage pairs that show asymmetric cross-resistance, and light grey for no observed cross-resistance.
(EPS)

S10 Fig Growth curves showing the fitness impact of resistance mutations. Growth curves of all 263 spontaneous resistant mutants grouped by focal phage resistance (up to 10 mutants per focal phage) and the wild-type ancestor (PAO1).
(EPS)

S11 Fig Frequency histograms of growth parameters for PAO1 and resistant mutants. Frequency histograms of growth parameters extracted from the bacterial growth curves (S10 Fig): maximum absorbance reached in 24 h (OD600), lag period calculated as x-axis intercept (time, hours) of a tangent to the growth curve at the point of maximum growth rate, maximum growth rate, and integral of growth curve. Red histogram shows the wild-type PAO1 ancestor, and blue shows 263 spontaneous resistant mutants.
(EPS)

S12 Fig Correlation of growth parameters. Multiple linear regression of growth parameters for 263 spontaneous resistant mutants (S11 Fig): the diagonal shows the distribution of each growth parameter, the bottom quadrant plots correlations for each pair of growth parameters, and the top quadrant gives corresponding R^2^ values.
(EPS)

S1 Table Mutations identified by whole-genome sequencing of spontaneous resistant mutants. Those highlighted in blue show mutations providing between-module cross-resistance.
(PDF)

S2 Table Overview of phage strains. Details of the isolation and characterisation of the phage collection.
(PDF)

S3 Table RAPD PCR primers. Primers used in the RAPD PCR analysis of phage genetic relatedness. RAPD, random amplification of polymorphic DNA.
(PDF)

S1 Data Growth data for bacterial cultures grown with or without phage used to calculate RBG. (TXT)

S2 Data Growth data for bacterial cultures used to calculate relative fitness. (TXT)

S3 Data RAPD PCR banding patterns for the phages used in this study. (TXT)

S4 Data Plaque forming units of the phages when plated onto the piliated wild-type PAO1 or the unpiliated pilB transposon mutant. (TXT)

S5 Data Resistance mutation frequency for PAO1 against individual and pairwise combinations of phages. (TXT)

S6 Data Growth data for bacterial cultures grown with or without indivdual or pairwise combinations of phages. (TXT)

## Abbreviations

CF: cystic fibrosis
CRF: cross-resistance frequency
CRI: cross-resistance index
CRISPR: clustered regularly interspaced short palindromic repeat
LPS: lipopolysaccharide
RAPD: random amplification of polymorphic DNA
RBG: relative bacterial growth
