# Supplementary material for: Cross-resistance is modular in bacteria–phage interactions
Source: PLoS Biol. 2018 Oct 3;16(10):e2006057. doi: 10.1371/journal.pbio.2006057 (PMC6188897; doi:10.1371/journal.pbio.2006057)
Supplement: S1 Table — Those highlighted in blue show mutations providing between-module cross-resistance. (PDF) [file pbio.2006057.s013.pdf]

Function	Gene mutated	Operon/Cluster	Gene product activity	Chromosome position (bp)	Effect type	Putative impact	Clone	Focal phage resistance	Cross-resistance provided
**Lipopolysaccharide biosynthesis**	wbpL	wbp cluster	Glycosyl transferase required for LPS A- and B-band biosynthesis	3529218	frameshift (1bp insertion)	HIGH	SM13.1_5	PA13P1	within module 2
	wzy	wbp cluster	LPS B-band O-antigen polymerase	3538988	frameshift (1bp insertion)	HIGH	SM10.2_1	PA10P2	within module 2
				3538988	frameshift (1bp insertion)	HIGH	SM14/1_3	14/1	within module 2
				3538988	frameshift (1bp insertion)	HIGH	SM2.1_3	PA2P1	within module 2
				3538988	frameshift (1bp insertion)	HIGH	SM5.1_8	PA5P1	within module 2
				3538988	frameshift (1bp insertion)	HIGH	SM7.2_6	PAP7P2	within module 2
**Type IV pilus motility**	pilB		Motor protein powering pilus extension	5071434	stop gained	HIGH	SMPNM_2	PNM	within module 1
				5070526	frameshift (4bp deletion)	HIGH	SMPNM_3	PNM	within module 1
	pilT		Motor protein powering pilus retraction	437147	missense variant	MODERATE	SM5.2_2	PA5P2	within module 1
				437058-438250	deletion (1,192bp)	HIGH	SM5.2_7	PA5P2	within module 1
				437130	disruptive inframe deletion (12bp)	MODERATE	SMPT7_1	PT7	within module 1
	pilY1	fimU-pilV-pilW-pilX-pilY1-pilY2-pilE operon	Putative anti-retraction factor	5101300	frameshift (1bp deletion)	HIGH	SMPNM_9	PNM	within module 1
	pilJ	pilJ-pilK-chpA-chpB-chpC-chpD operon	Transduction of chemotaxis-related signals to two-component system regulating type IV pilus motility	453128	missense variant	MODERATE	SMPT7_5	PT7	within module 1
**Type IV pilus biosynthesis**	**pilD**	**pilC-pilD-coaE-PA4530 operon**	**N-methyltransferase involved in processing prepilins**	**5073276**	**stop gained**	**HIGH**	**SM5.2_9**	**PA5P2**	**between modules**
	pilQ	pilM-pilN-pilO-pilP-pilQ operon	Outer membrane secretin pore within pilus structure	5676479-5676662	deletion (183bp)	HIGH	SM5.2_6	PA5P2	within module 1
	fimV	fimV-truA-trpF operon	Peptidoglycan binding protein involved in pilus assembly	3497951-3497986	frameshift (35bp deletion)	HIGH	SMPNM_6	PNM	within module 1
**Signaling and global regulation**	**rpoN**	**rpoN-PA4463-ptsN-PA4465 -PA4466 operon**	**Alternative RNA polymerase sigma-54 factor (global gene regulation); regulates expression of pilA, which encodes the major pilin structural protein**	**4993096**	**frameshift (1bp deletion)**	**HIGH**	**SMPNM_8**	**PNM**	**between modules**
				**4994077**	**missensevariant**	**MODERATE**	**SMPT7_7**	**PT7**	**between modules**
	pilR	pilS-pilR operon	Two component system: PilS (sensor) and PilR (regulator) regulate production of the major pilin protein, PilA, through RpoN	5096180	frameshift(1bpdeletion)	HIGH	SM5.2_3	PA5P2	within module 1
				5096282	missensevariant	MODERATE	SMphiKZ_5	Φ KZ	within module 1
	**pilS**			**5094532**	**stopgained**	**HIGH**	**SMPT7_9**	**PT7**	**between modules**
**Other**	PA1875	PA1874-PA1875-PA1876 -PA1877 operon	Putative efflux pump	2045090	missensevariant	MODERATE	SMPT7_5	PT7	within module 1
