# Supplementary material for: Cross-resistance is modular in bacteria–phage interactions
Source: PLoS Biol. 2018 Oct 3;16(10):e2006057. doi: 10.1371/journal.pbio.2006057 (PMC6188897; doi:10.1371/journal.pbio.2006057)
Supplement: S2 Table — Details of the isolation and characterisation of the phage collection. (PDF) [file pbio.2006057.s014.pdf]

Strain	Source	Date first isolated	Isolation Host	Morphotype	Genome size (kb)	Accession number
**ΦKZ**	Sewage water, Kazakhstan	1975		Myoviridae	280.33	NC_004629.1
**PNM**	Mtkvari River, Tbilisi, Georgia	1999		Podoviridae	42.72	-
**PT7**	Lake Ku, Tibilisi, Georgia	1999		Myoviridae	58.2	-
**14/1**	Sewage water, Regensburg, Germany	2000		Myoviridae	66.23	NC_011703.1
**PA1P1**	Sewage water, Jyväskylä, Finland	2015	*Pseudomonas aeruginosa* 61841	-	-	-
**PA1P2**			*Pseudomonas aeruginosa* 61841	-	-	-
**PA1P3**			*Pseudomonas aeruginosa* 61841	-	66.46	-
**PA1P4**			*Pseudomonas aeruginosa* 61841	-	-	-
**PA1P5**			*Pseudomonas aeruginosa* 61841	-	-	-
**PA2P1**			*Pseudomonas aeruginosa* 61823	-	-	-
**PA4P2**			*Pseudomonas aeruginosa* 61432	-	-	-
**PA5P1**			*Pseudomonas aeruginosa* 11AN03663	-	-	-
**PA5P2**			*Pseudomonas aeruginosa* 11AN03663	-	-	-
**PA7P1**			*Pseudomonas aeruginosa* 62314	-	-	-
**PA7P2**			*Pseudomonas aeruginosa* 62314	-	-	-
**PA8P1**			*Pseudomonas aeruginosa* 62263	-	-	-
**PA8P2**			*Pseudomonas aeruginosa* 62263	-	-	-
**PA10P1**			*Pseudomonas aeruginosa* 62206	-	-	-
**PA10P2**			*Pseudomonas aeruginosa* 62206	-	-	-
**PA10P3**			*Pseudomonas aeruginosa* 62206	-	-	-
**PA11P1**			*Pseudomonas aeruginosa* 62180	-	66.06	-
**PA11P2**			*Pseudomonas aeruginosa* 62180	-	-	-
**PA12P1**			*Pseudomonas aeruginosa* 62181	-	-	-
**PA12P2**			*Pseudomonas aeruginosa* 62181	-	-	-
**PA13P1**			*Pseudomonas aeruginosa* 62172	-	-	-
**PA13P2**			*Pseudomonas aeruginosa* 62172	-	-	-
**PA14P2**			*Pseudomonas aeruginosa* 62109	-	-	-
