# Supplementary material for: Cross-resistance is modular in bacteria–phage interactions
Source: PLoS Biol. 2018 Oct 3;16(10):e2006057. doi: 10.1371/journal.pbio.2006057 (PMC6188897; doi:10.1371/journal.pbio.2006057)
Supplement: S3 Table — Primers used in the RAPD PCR analysis of phage genetic relatedness. RAPD, random amplification of polymorphic DNA. (PDF) [file pbio.2006057.s015.pdf]

Name	Sequence	Reference
RAPD5[i]/Primer5[ii]	5'AACGCGCAAC3'	[i.] [1] [ii.] [2]
P1	5'CCGCAGCCAA3'	[1]
P2	5'AACGGGCAGA3'	[1]
OPL5	5'ACGCAGGCAC3'	[1]
Primer1	5'GGTGCGGGAA3'	[2]
Primer2	5'GTTTCGCTCC3'	[2]
Primer3	5'GTAGACCCGT3'	[2]
Primer4	5'AAGAGCCCGT3'	[2]
Primer6	5'CCCGTCAGCA3'	[2]

1. AzizianR,NasserA,AskariH,TaheriKalaniM,SadeghifardN,PakzadI,etal.Sewageasa richsourceofphagestudyagainstPseudomonasaeruginosaPAO.Biologicals.2015;43: 238–241.doi:10.1016/j.biologicals.2015.05.004

2. KumariS,HarjaiK,ChhibberS.Isolationandcharacterizationof *Klebsiellapneumoniae* specificbacteriophagesfromsewagesamples.FoliaMicrobiol(Praha).2010;55:221 – 227.doi:10.1007/s12223 -010-0032-7
